# Green and Innovative Extraction: Phenolic Profiles and Biological Activities of Underutilized Plant Extracts Using Pulsed Electric Fields and Maceration

**DOI:** 10.3390/foods14020222

**Published:** 2025-01-13

**Authors:** Noelia Pallarés, Houda Berrada, Emilia Ferrer, Wahiba Rached, José Pinela, Filipa Mandim, Tania C. S. P. Pires, Tiane C. Finimundy, Francisco J. Barba, Lillian Barros

**Affiliations:** 1 Department of Preventive Medicine and Public Health, Food Science, Toxicology and Forensic Medicine, Faculty of Pharmacy, Universitat de València, Avda. Vicent Andrés Estellés, Burjassot, 46100 València, Spain; noelia.pallares@uv.es (N.P.); houda.berrrada@uv.es (H.B.); emilia.ferrer@uv.es (E.F.); rachedwahibadz@gmail.com (W.R.); francisco.barba@uv.es (F.J.B.); 2 Centro de Investigação de Montanha (CIMO), Laboratório Associado para a Sustentabilidade e Tecnologia em Regiões de Montanha (SusTEC), Instituto Politécnico de Bragança, Campus de Santa Apolónia, 5300-253 Bragança, Portugal; jpinela@ipb.pt (J.P.); filipamadim@ipb.pt (F.M.); tania.pires@ipb.pt (T.C.S.P.P.); lillian@ipb.pt (L.B.); 3 National Institute for Agricultural and Veterinary Research (INIAV), I.P., Rua dos Lágidos, Lugar da Madalena, Vairão, 4485-655 Vila do Conde, Portugal; 4 Nutrition and Bromatology Group, Department of Analytical Chemistry and Food Science, Faculty of Science, Universidad de Vigo, 32004 Ourense, Spain

**Keywords:** pulsed electric fields, maceration, Algerian medicinal plants, phenolic compounds, biological activities

## Abstract

Underutilized plant species such as *Asteriscus graveolens* (Forssk.) Less., *Haloxylon scoparium* Pomel, and *Ruta chalepensis* L. have been historically valued in traditional medicine for their potential health benefits. These species present an untapped source of bioactive compounds with significant applications in the food and pharmaceutical industries, including the development of functional foods and additives. Recent advances in food processing have introduced innovative methods, such as pulsed electric fields (PEFs), to enhance the extraction of valuable compounds without compromising their integrity or quality. This study investigates the impact of PEF technology on the recovery of bioactive compounds from these plants, comparing it with conventional maceration (MAC) techniques. Phenolic compound profiles and biological activities, including antioxidant, antimicrobial, anti-inflammatory, and cytotoxic effects, were evaluated. The results demonstrated that for *R. chalepensis*, PEF extraction achieved comparable phenolic content (58 mg/g) to MAC (72 mg/g). However, MAC generally provided higher phenolic yields for other plants. *A. graveolens* extracts exhibited significant antitumoral and anti-inflammatory potentials. The antimicrobial results indicated that MAC extracts were more effective against bacterial growth, while PEF extracts outperformed MAC against *A. brasiliensis* (MIC: 10 mg/mL). Antioxidant potential was observed in both methods, with TBARS IC_50_ values ranging from 17 to 79.5 µg/mL. While MAC generally yielded superior results, PEF extraction showed great promise as an environmentally sustainable alternative, eliminating the need for organic solvents and aligning with green extraction principles.

## 1. Introduction

In recent years, global challenges such as climate change, geopolitical conflicts, and the ongoing quest for sustainable development have highlighted the need for more efficient and eco-friendly production models. One promising strategy is the utilization of local and underexploited natural resources, which have significant potential for various applications [1,2]. Medicinal and edible plants, for instance, offer a wealth of bioactive compounds that can be harnessed as food additives, pharmaceutical agents, or industrial components [3,4,5].

Underutilized plant species, including *Asteriscus graveolens* (Forssk.) Less., *Haloxylon scoparium* Pomel, and *Ruta chalepensis* L. are indigenous to the Mediterranean and Near East regions. These plants have been traditionally employed in folk medicine to address a wide range of conditions, such as gastric disorders, diabetes, rheumatism, and various inflammatory and infectious diseases [6,7,8]. Additionally, they have been used in the preparation of infusions, dietary supplements, essential oils, and cosmetics [9]. Research indicates that these species contain valuable compounds such as polysaccharides, essential oils, alkaloids, flavonoids, and saponins, which exhibit antioxidant, anti-inflammatory, antibacterial, and anticancer activities. These attributes make them promising candidates for the prevention and treatment of several diseases, including neurological, cardiovascular, and inflammatory disorders [10,11]. Furthermore, the functional potential of these compounds has garnered interest from researchers and industries alike for the development of innovative food products and bioactive ingredients [12,13].

The extraction of bioactive compounds from plants remains a significant challenge, particularly due to the need to preserve the integrity of these compounds. Traditional extraction methods, while widely used, often require long processing times, extensive use of organic solvents, and high energy consumption [14]. Emerging extraction technologies have gained prominence to address these limitations. Among these, pulsed electric field (PEF) technology, based on the phenomenon of electroporation, stands out as a green, cost-effective, and efficient alternative. PEFs involve applying electric fields of varying strengths (0.1–40 kV/cm) over short durations, leading to reduced energy use and high-quality extracts [15]. This technology is particularly well-suited for sustainable production processes, aligning with the increasing demand for environmentally friendly approaches [16,17,18,19,20]. PEF technology enables the recovery of bioactive compounds, such as macronutrients, polyphenols, and pigments from food by-products and waste using low energy. However, the effectiveness of the treatment can be affected by the parameters of PEF equipment and external factors such as pH, conductivity, and concentration of the treated solution. Therefore, the extraction mechanisms of PEF treatment should be verified, and extraction kinetics models should be developed and tested. It is also necessary to optimize the geometry of the PEF treatment chambers and scale up the extraction technology for better use in industrial applications [21].

This study aims to evaluate the efficiency of maceration (MAC) and pulsed electric field (PEF) extraction methods for obtaining bioactive compounds from the underexploited plants *A. graveolens*, *H. scoparium*, and *R. chalepensis*, highlighting their potential for industrial and medicinal applications.

## 2. Materials and Methods

### 2.1. Chemicals and Reagents

Standards for phenolic compounds were acquired from Sigma (St. Louis, MO, USA). Essential cell culture materials were sourced from Hyclone (Logan, UT, USA). Bacterial and fungal media were obtained from Biolab^®^ (Budapest, Hungary), and blood agar with 7% sheep blood was supplied by LiofilChem S.r.l (Roseto d’Abruzzi, Italy).

Antibiotics were provided by Fisher Scientific (Janseen Pharmaceutical, Belgium), while the antifungal agent was sourced from Frilabo (Porto, Portugal). Additional chemicals used in the study included trichloroacetic acid (TCA), tris(hydroxymethyl) aminomethane (Tris), lipopolysaccharide (LPS), ascorbic acid, sodium nitrate, Griess reagent system (Promega), sulphorodamine B (SRB), and dimethyl sulfoxide (DMSO), all obtained from Sigma-Aldrich (St. Louis, MO, USA). Panreac Applichem (Barcelona, Spain) provided p-iodonitrotetrazolium chloride (INT), thiobarbituric acid (TBA), and sodium sulfate, while ACROS Organics (Geel, Belgium) supplied iron (II) sulfate. Solvents, HPLC grade, were procured from Fisher Scientific (Leicestershire, UK), and antioxidants, including Trolox (6-hydroxy-2,5,7,8-tetramethylchroman-2-carboxylic acid) and 2,2′-azobis(2-methylpropionamidine) dihydrochloride (AAPH), were also acquired from Sigma (St. Louis, MO, USA).

### 2.2. Plant Material

The leaves of *Asteriscus graveolens* (Forssk.) Less. were gathered in October 2019 from Bayed, located in the southwest of Algeria. The aerial parts of *Haloxylon scoparium* Pomel were collected in June 2020 from Naâma, also in southwestern Algeria, while the aerial parts of *Ruta chalepensis* L. were harvested in June 2021 from Oran, in the western region of Algeria. Following collection, the plant materials were air-dried, ground into a fine powder, and stored in a dry, dark environment to preserve their quality until further analysis.

### 2.3. Samples Extraction

#### 2.3.1. Maceration

To perform maceration (MAC), 1.5 g of plant material was extracted using a solution of ethanol and water (EtOH:H2O, 80:20 *v*/*v*; 30 mL) at room temperature with constant magnetic stirring for 1 h. The resulting solution was filtered using Whatman No. 4 filter paper and collected. The extraction process was repeated and was concentrated under reduced pressure at 40 °C using a rotary evaporator (Heidolph). The remaining aqueous fraction was subsequently frozen and freeze-dried using a FreeZone 4.5 lyophilizer (Labconco, Kansas City, MO, USA). The final extracts were stored in a dark, protected environment until further analysis.

#### 2.3.2. Pulsed Electric Fields

For each plant species, 4 g of material was mixed with 200 mL of water to create a 2% (*w*/*v*) aqueous suspension. This suspension was then processed using pulsed electric field (PEF) technology, applied via an Elea Cellcrack III system (German Institute of Food Technologies, Quakenbrück, Germany). The extraction parameters included in the study were fixed at a voltage of 30 kV, resulting in a field strength of 3 kV/cm and a specific energy of 100 kJ/kg. Temperature and conductivity measurements of the sample were taken both before and after treatment using a ProfiLine Cond 3310 portable conductivity meter (WTW, Xylem Analytics, Weilheim in Oberbayern, Germany). Following PEF treatment, the mixture was stirred for 1 h and vacuum-filtered. The resulting filtrate was centrifuged at 5000 rpm for 10 min, and the supernatant was frozen and subsequently freeze-dried using a Telstar LyoQuest-55 freeze dryer (Syntegon Telstar, Barcelona, Spain).

### 2.4. Chemical Characterization

To analyze the phenolic compounds, 10 mg of the freeze-dried plant extracts were dissolved in a mixture of ethanol and water (EtOH:H_2_O, 20:80 *v*/*v*) to achieve a final concentration of 10 mg/mL. The samples were filtered using 0.2 µm filters and analyzed utilizing a Dionex Ultimate 3000 UPLC system (Thermo Scientific, San Jose, CA, USA), which was equipped with a diode array detector (DAD), and an electrospray ionization mass spectrometer (LC-DAD-ESI/MS^n^). The setup included an auto-sampler maintained at 5 °C, a degasser, a column compartment with controlled temperature, and a quaternary pump. Chromatographic separation was performed using a Waters Spherisorb S3 ODS-2C18 column (3 μm, 4.6 mm × 150 mm, Waters, Milford, MA, USA) maintained at 35 °C. The mobile phases consisted of 0.1% formic acid in water (A) and acetonitrile (B). The elution gradient began at 15% B for 5 min, increased to 20% B over the next 5 min, followed by 25% B in 10 min, 35% B in another 10 min, and finally 50% B in 10 min, before re-equilibration. The flow rate was maintained at 0.5 mL/min. Detection was performed at 280, 330, and 370 nm using the DAD and in negative ion mode using a Linear Ion Trap LTQ XL mass spectrometer (Thermo Finnigan, San Jose, CA, USA) with an ESI source. Operational settings included a sheath gas pressure of 50 psi, a spray voltage of 5 kV, a source temperature of 325 °C, and a capillary voltage of −20 V, with a tube lens offset at −66 V. Mass spectra were acquired over an *m*/*z* range of 100 to 1500 using Xcalibur^®^ 4.2.47 software (Thermo Finnigan, Waltham, MA, USA).

The phenolic compounds were quantified using commercial standards with calibration curves. In cases where standards were unavailable, tentative identification relied on UV spectra, fragmentation patterns, and retention times. The calibration curves, detection limits (LODs), and quantification limits (LOQs) for each standard are summarized in Table 1. All measurements were performed in triplicate, with results expressed in mg per g of dry extract.

### 2.5. In Vitro Biological Activities

#### 2.5.1. Cytotoxicity

The cytotoxic potential was tested in different cell lines. The human tumor cells of colorectal adenocarcinoma (CaCO_2_), gastric adenocarcinoma (AGS), breast adenocarcinoma (MCF-7), and lung carcinoma (NCI-H460) were tested. African green monkey kidney (Vero) and a primary culture established from pig liver (PLP2) were the non-tumoral cell lines analyzed. All cell lines, except the Vero cell line, were routinely maintained in RPMI-1640 medium supplemented with 10% fetal bovine serum, glutamine (2 mM), penicillin (100 U/mL), and streptomycin (100 mg/mL). All cell lines were maintained in culture flasks in the incubator under a humid atmosphere at 37 °C and 5% CO_2_, and the medium was replaced every two days. For the experiments, the cells were used when a 70 to 80% confluence was obtained. To test the cytotoxic potential, serial dilutions were prepared (0.125–8 mg/mL). An aliquot of each extract concentration (10 μL; final concentrations tested 6.25–400 µg/mL) was incubated with 190 μL of cell suspension. The 96-well microplates were incubated for 72 h at the same conditions specified above.

After the incubation period, the cells were fixed with trichloroacetic acid (TCA) (10% *w*/*v*; 100 μL) previously cooled at 4 °C. Then, the plates were incubated for 1 h at 4 °C, washed three times with water, and dried. After, 100 μL of sulforhodamine B (SRB) solution at 0.057% (*w*/*v*) was added, and the plates were left for 30 min at room temperature. Subsequently, the plates were washed three times with a solution of acetic acid (1%; *v*/*v*) to remove non-adhered SRB and let it dry. Finally, the adhered SRB was solubilized by adding 200 μL of Tris (10 mM), and the absorbance was measured at 540 nm (ELX800 Biotek microplate reader, Bio-Tek Instruments, Inc., Winooski, VT, USA). The results were expressed in terms of the concentration of plant extracts with the ability to inhibit 50% of cell proliferation (GI_50_; µg/mL). Ellipticin was employed as a positive control.

#### 2.5.2. Anti-Inflammatory Activity

The anti-inflammatory activity of the plant extracts was carried out according to the methodology previously described by [22]. The RAW 264.7 mouse macrophage cell line was commercially obtained from Institute DMSMZ (German Collection of Microorganisms and Cell Cultures GmbH) and was maintained with DMEM medium supplemented as described above and maintained in an incubator according to the same conditions reported in Section 2.5.1. RAW 264.7 cells were detached with a cell scraper, and then 300 μL of the cell suspension (500,000 cells/mL) was transferred to 96-well microplates. After their incubation for 24 h, the medium was replaced with 150 μL of fresh medium, and the cells were exposed to the tested concentrations of extracts (15 μL; 6.25–400 μg/mL) prepared as indicated above and incubated for 1 h. After the incubation period, a lipopolysaccharide solution (LPS; 1 mg/mL) was added to the wells. After 24 h of incubation, the nitric oxide concentration was determined through a colorimetric reaction with a Griess reagent system kit. The absorbance was read at 540 nm (ELX800 Biotek microplate reader, Bio-Tek Instruments, Inc., Winooski, VT, USA), and nitric oxide was quantified by a nitrite calibration curve. The results were obtained through the graphical representation of the percentage of inhibition of nitric oxide production versus the plant extracts concentration and expressed in relation to the concentration of each of the plant extracts responsible for 50% inhibition of nitric oxide production (IC_50_; µg/mL).

#### 2.5.3. Antibacterial Activity

The antibacterial activity of plant extracts was tested against eight foodborne bacteria, five Gram-negative (*Enterobacter cloacae* (ATCC 49741)—*Escherichia coli* (ATCC 25922), *Pseudomonas aeruginosa* (ATCC 9027), *Salmonella enterica subsp* (ATCC 13076), *Yersinia enterocolitica* (ATCC 8610))—and three Gram-positive (*Bacillus cereus* (ATCC 11778)—*Listeria monocytogenes* (ATCC 19111) and *Staphylococcus aureus* (ATCC 25923)). All bacteria were purchased at Frilabo, Porto, Portugal, and were incubated at 37 °C in an appropriate fresh medium for 24 h before the experiments to maintain their exponential growth phase.

The minimum inhibitory concentration (MIC) was determined using a colorimetric assay adapted from the method outlined [23]. Lyophilized plant extracts (50 mg) were dissolved in a DMSO:H2O solution (5%, *v*/*v*) to prepare a stock concentration of 20 mg/mL. The stock solution was then serially diluted with a Tryptic Soy Broth (TSB) medium to achieve final test concentrations ranging from 10 to 0.03125 mg/mL. To each well of a microplate, 10 μL of bacterial inoculum was added. The plates were incubated at 37 °C for 24 h. Following this, 40 μL of p-iodonitrotetrazolium chloride (INT) solution (0.2 mg/mL) was added, and the plates were incubated for an additional 30 min at 37 °C. MIC values were defined as the lowest concentration of plant extract that inhibited a color change from yellow to pink, indicating bacterial growth inhibition.

To assess the minimum bactericidal concentration (MBC), 10 μL from wells with concentrations higher than the MIC were transferred onto solid blood agar plates (7% sheep blood) and incubated at 37 °C for 24 h. The MBC was recorded as the lowest concentration of extract that completely inhibited bacterial colony formation.

#### 2.5.4. Antifungal Activity

Two fungal species were used to test the antifungal capacity of plant extracts: *Aspergillus fumigatus* (ATCC 204305) and *Aspergillus brasiliensis* (ATCC 16404). The fungal strains were purchased from Frilabo, Porto, Portugal. The micromycetes were maintained on malt agar, and the cultures were stored at 4 °C. Then, the fungal species were placed and incubated with a new medium at 25 °C for 72 h. To count the number of spores, the fungal spores were washed from the surface of agar plates employing sterile saline (0.85%) containing 0.1% Tween 80 (*v*/*v*), then the spore suspension was adjusted with sterile saline to reach an approximate concentration of 1.0 × 10^5^ in the plate. The freeze-dried plant extracts were reconstituted following the previously described method (Section 2.5.3) and subsequently diluted with Malt Extract Broth (MEB) to obtain the final concentrations tested (10 to 0.03125 mg/mL). The MIC values were determined using a serial dilution method. To assess the minimum fungicidal concentration (MFC), 2 μL of the tested compounds, prepared in the appropriate medium, were placed into microplates, each well containing 100 μL of Malt Extract Broth (MEB), and incubated for 72 h. The MFC was determined as the minimum concentration that completely inhibited visible fungal growth. Ketoconazole (Frilabo, Porto, Portugal) was used as a positive control to validate the assay.

#### 2.5.5. Antioxidant Activity

The antioxidant capacity of the studied samples was evaluated using two cell-based assays: the thiobarbituric acid reactive substance assay (TBARS) and the oxidative hemolysis inhibition assay (OxHLIA).

The TBARS method is based on the detection of malondialdehyde (MDA), the major lipid oxidation product, and was conducted according to the methodology previously reported [24]. The stock solution in a concentration of 10 mg/mL of the studied plant extracts was prepared. The range of concentrations to be tested was obtained by serial dilutions (0.625–0.010 mg/mL). The extracts were incubated with the brain tissue homogenate prepared with Tris HCl buffer (20 mM, pH 7.4; 1:2, *w*/*v*), ascorbic acid (100 μL), and ferrous sulfate (100 μL) for 1 h at 37.5 °C. After the incubation period, a solution of trichloroacetic acid (500 µL; 28%, *w*/*v*), followed by 380 µL of thiobarbituric acid (2%, *w*/*v*), was added. The mixture was incubated at 80 °C for 20 min. Then, the samples were centrifuged at 3000× *g* for 10 min, and the supernatant was transferred to 96-well microplates to measure the color intensity at 532 nm (Synergy HTX Bioteck^®^). Trolox was used as a positive control, and the solvents water and Tris without extracts were used as a negative control. The inhibition percentage (%) was obtained using the following formula: inhibition percentage (%) = [(A − B)/A] × 100%, where A and B are the absorbances of the control and the sample solution, respectively. The results were expressed as the extract concentration responsible for 50% lipid peroxidation inhibition (IC_50_).

The antihemolytic activity was evaluated by the authors of [25]. Briefly, 200 µL of a sheep erythrocyte solution (2.8%, *v*/*v*) prepared in phosphate-buffered saline (PBS, pH 7.4) was mixed with 400 µL of (i) plant extracts redissolved in PBS (0.125–8 mg/mL), (ii) the positive control Trolox (7.81–250 µg/mL in PBS), and (iii) PBS (negative control) or distilled water (for complete hemolysis). After 10 min incubation at 37 °C, 200 μL of the free-radical generator 2.2′-azobis(2-methylpropionamidine) dihydrochloride (AAPH, 160 mM in PBS) was added, and the optical density was kinetically measured at 690 nm in a microplate reader (Bio-Tek Instruments Inc., ELX 800, Winooski, VT, USA) every 10 min until complete hemolysis. The results were expressed as the extract concentration required to inhibit hemolysis by 50% (IC_50_; µg/mL) for a period of 60 min.

### 2.6. Statistical Analysis

All experiments were conducted in triplicate. Statistical analyses were carried out using GraphPad Prism version 8.0.2 (GraphPad Software, San Diego, CA, USA). Data were analyzed using one-way analysis of variance (ANOVA) to evaluate significant differences between groups, followed by Tukey’s Honestly Significant Difference (HSD) test for post hoc comparisons. A significance threshold of *p* < 0.05 was applied. Results are presented as the mean ± standard deviation (SD). For comparisons involving only two groups, Student’s *t*-test was utilized.

## 3. Results and Discussion

### 3.1. Phenolic Compounds Identification

Table 1 summarizes the chromatographic properties (retention time, λmax, pseudomolecular ions, and MS^2^ fragments) and the tentative identification of each phenolic compound. Individual phenolic compounds were identified by analyzing the data and comparing them with established standards and information from prior studies in the literature. For *A. graveolens*, nine phenolic compounds were identified, among which seven were classified as flavonoids and two as phenolic acids. Among the flavonoids, **Peaks 2**, **3**, and **9** were identified as flavones, tentatively assigned as trihydroxyflavone (apigenin-di-*C*-glucoside), tetrahydroxyflavone (luteolin-*O*-acetylhexoside), and *O*-methylated flavone (tricin-*O*-glucoside), respectively. Additionally, three quercetin derivatives linked to sugars were also identified in this species, namely quercetin-3-*O*-rutinoside (**Peak 5**), quercetin-3-*O*-glucuronide (**Peak 6**), and quercetin-*O*-malonylglucoside (**Peak 8**). An eriodictyol glycoside derivative (**Peak 4**) was also identified, considering the maximum absorption and fragmentation pattern of the compound, being tentatively identified as eridictyol-*O*-hexoside. Two phenolic acids were also detected, being identified as 5-*O*-caffeoylquinic acid (**Peak 1**) and 3,5-*O*-dicaffeoylquinic acid (**Peak 7**), by comparing the chromatographic characteristics with commercial standards, and the latter one with previously described compounds identified by [26,27]. Previous studies on the phenolic composition of *A. graveolens* [28,29] have identified the presence of various compounds, including derivatives of kaempferol and quercetin, across different extracts of this species.

For *R. chalepensis*, a total of 17 phenolic compounds were identified, comprising 8 phenolic acids and 9 flavonoids. Among these, **Peaks 10** and **11** were tentatively identified as 4-**p**-coumaroylquinic acid and 4-*O*-feruloylquinic acid, respectively [25]. **Peaks 15** and **22–25** were tentatively identified as sinapoyl derivatives linked to a disaccharide, namely gentiobioside. Regarding flavonoids, three *C*-glycosylated apigenin derivatives represented by **Peaks 12**, **13,** and **14** were tentatively identified as apigenin-6-*C*-glucose-8-*C*-glucose, apigenin-8-*C*-glucose-6-*C*-glucose, and apigenin-*O*-glucuronyl-hexoside, respectively, and were previously described by [30]. Quercetin derivatives were also found; **Peak 16** was tentatively identified as quercetin-*O*-deoxihexosyl-hexoside, being previously reported in *R. graveolens* samples by [30]. **Peak 18** was identified as quercetin-3-*O*-rutinoside, and **Peak 19** was tentatively identified as quercetin-3-*O*-glucuronide, previously described by [31] in *R. graveolens* leaves. Finally, **Peaks 17** and **21** were tentatively identified as isorhamnetin-*O*-di-hexoside and isorhamnetin-3-*O*-rutinoside, respectively, and were previously described by [32].

In *H. scoparium*, a total of 18 phenolic compounds were identified, including 6 phenolic acids and 12 flavonoids. Among the flavonoids, flavonols were the only subgroup detected, with isorhamnetin glycoside derivatives being particularly prominent. **Peak 27** was confirmed as protocatechuic acid through comparison with commercial standards. **Peaks 28, 31**, and **33** were characterized as caffeic acid, caffeic acid acetylhexoside, and caffeic acid-*O*-(sinapoyl-*O*-hexoside), respectively, based on their mass spectra and MS fragmentation data. **Peaks 29** and **30** were identified as glycosyringic acid and *p*-coumaric acid hexoside. Several peaks were attributed to isorhamnetin derivatives based on their UV and mass spectral characteristics. **Peaks 32**, **34–36,** and **38–44** were identified as isorhamnetin glycoside derivatives, showing λmax values around 348–356 nm and pseudomolecular ions releasing an MS^2^ fragment at *m*/*z* 315, consistent with isorhamnetin. **Peaks 43** and **44** were specifically identified as isorhamnetin-3-*O*-rutinoside and isorhamnetin-3-*O*-glucoside. Other isorhamnetin derivatives were tentatively identified using pseudomolecular ions and fragmentation patterns: isorhamnetin-*O*-hexoside-*O*-deoxyhexosyl-hexoside (**Peak 32**), isorhamnetin-*O*-hydroxyferuloylhexoside-*O*-malonylhexoside isomers I and II (**Peaks 34** and **35**), isorhamnetin-*O*-di-hexoside (**Peak 36**), isorhamnetin-*O*-di-deoxyhexosyl-hexoside (**Peak 38**), isorhamnetin-*O*-deoxyhexosyl-pentosyl-hexoside isomers I and II (**Peaks 39** and **40**), and isorhamnetin-*O*-deoxyhexoside-*O*-hexoside isomers I and II (**Peaks 41** and **42**). **Peak 37** was tentatively identified as a quercetin-*O*-pentosyl-deoxyhexosyl-hexoside.

Table 2 presents the quantification of phenolic compounds identified in the MAC and PEF extracts. In general, maceration proved to be a more effective method for extracting phenolic compounds. Among these, *A. graveolens* extracts revealed a total phenolic compound content practically double when obtained through MAC (30.7 mg/g) in comparison to PEF (13.6 mg/g) extracts. More concretely, total phenolic acids were quantified as 22.8 mg/g of extract for the maceration technique and 6.6 mg/g of extract in the PEF extraction methodology. Among phenolic acids, 5-*O*-caffeoylquinic acid was the predominant: 19.3 mg/g (maceration extract) vs. 5.83 mg/g (PEF extract). However, the content in total flavonoids was similar after comparing both extracts: 7.9 mg/g (MAC extract) vs. 7.0 mg/g (PEF extract). The most predominant flavonoid in the MAC extract was quercetin-*O*-glucuronide (4.9 mg/g), while in the PEF extract, the main compound was quercetin-*O*-malonylhexoside (2.6 mg/g) (Table 2). In a prior study, chromatographic analysis identified a range of phenolic compounds in *A. graveolens* samples, including one phenolic acid, trans-cinnamic acid, and eleven flavonoids. The flavonoids predominantly included myricetin and its *O*-glucoside derivative, luteolin and its glycosides (cynaroside, orientin, and iso-orientin), rutin (a diglycoside of quercetin), hyperoside (quercetin 3-*O*-galactoside), and apigenin and its *C*-glycosylated derivatives (vitexin and iso-vitexin). These authors suggested that the potential pharmacological properties may be attributed to flavonoid compounds, being the most predominant group of phenolic compounds [33,34]. Some of these flavonoids were also identified in the present study. Although phenolic acids were predominant in MAC extract, flavonoids were the most abundant within the PEF extraction.

The phenolic profile of *R. chalepensis*, obtained employing both extraction techniques, was similar, obtaining 72 mg/g (MAC extract) vs. 58 mg/g (PEF extract). The total flavonoid content was quantified in 54 mg/g (MAC extract) and 46.5 mg/g (PEF extract), being the most predominant compound. Among phenolic acids, 4-p-coumaroylquinic acid was the main compound in both extracts. Total phenolic acids quantified in the MAC extract were 18 mg/g and 11.4 mg/g in the PEF extract (Table 2). In this case, the content of flavonoid compounds was higher than phenolic acids. In a previous study, 4-*O*-*p*-comaroylquinic acid and 4-*O*-feruloylquinic acid (phenolic acids) were also determined in hydroalcoholic *R. graveolens* extract, where only 13 phenolic compounds were determined [35,36,37].

Finally, for *H. scoparium*, important differences were observed when comparing the contents of MAC extract with PEF extract, with a total phenolic content of 144 mg/g vs. 33.8 mg/g. In this case, the content of flavonoid contents was 19.4 mg (MAC extract) vs. 12.5 mg/g (PEF extract), being isorhamnetin-*O*-hexoside-*O*-rutinoside, the most abundant flavonoid in MAC extract and isorhamentin-*O*-deoxyhexosyl-pentosyl-hexoside in PEF extract. Regarding phenolic acids, an important amount was determined in MAC extract (125 mg/g), which was 6× higher than that quantified in PEF extract (21.3 mg/g) (Table 2). The primary phenolic acid identified in the MAC extract was glycosyringic acid, which was absent in the PEF extract. This difference likely accounts for the significant variation in total phenolic acid content between the two extraction methods. In contrast, the main phenolic acid found in the PEF extract was p-coumaric acid hexoside. Both compounds have been previously reported in the literature in studies involving plants from the Amaranthaceae family [38,39]. In a previous study performed on *H. scoparium*, these phenolic acids were not detected, but caffeic acid and quercitin were identified, which were also observed in the present study [40]. In previous studies, an important content of phenolic compounds in *H. scoparium* hydroalcoholic extract was also found, with a total phenolic content of around 108.41 (mg/g, dry weight). In contrast, the reported content of flavonoids was lower, with total flavonols and flavones of 2.72 (mg/g, dry weight) [41]. The same trend was also observed in our study.

### 3.2. Biological Activities of Algerian Medicinal Plants Extracts

#### 3.2.1. Cytotoxic Activity

The results obtained from the studied plants (*A. graveolens*, *H. scoparium*, and *R. chalepensis*) are presented in Table 3. We observed the influence of the extraction methodology on the cytotoxic potential. In general, higher cytotoxic activities were observed for MAC extraction, except for *H. scoparium*, where PEF extracts showed a higher capacity to interfere with cell proliferation. Among all the extracts obtained, *A. graveolens* exhibited the highest cytotoxicity activity with the lowest GI_50_ values. Comparing extraction procedures, *A. graveolens* MAC extraction presented the highest cytotoxic activities (GI_50_ values between 13 and 56 µg/mL) compared to PEF extracts (GI_50_ values between 56 and 85 µg/mL) (Table 3). In the case of *H. scoparium*, similar cytotoxicity was observed for both extraction procedures applied, with GI_50_ values ranging from 146 to 275 µg/mL for the MAC extraction and 81 to 266 µg/mL for PEF extraction. For instance, in the Vero cell line, the GI_50_ values exhibited by *H. scoparium* PEF extract were significantly lower than those obtained for the MAC procedure (81 and 146 µg/mL, respectively). Finally, the results obtained for *R. chalepensis* PEF extracts did not show the capacity to inhibit cell proliferation at the tested concentrations (GI_50_ > 400 µg/mL), contrary to what was verified for the MAC (GI_50_ values between 39 and 136 µg/mL).

In a previous study, the cytotoxic properties of Algerian medicinal plants, including *A. graveolens* and *H. scoparium*, were investigated on the proliferation of four human tumor cell lines (MCF-7, NCI-H460, HeLa, and HepG2, and a non-tumor cell line (PLP2). The reported GI_50_ values (between 11 and >400 µg/mL) were similar to those reported in the present study [28]. Another Algerian plant, Tetraclinis articulata (Vahl) masters leaves, employed for medicinal purposes, also showed promising cytotoxic properties. In this case, the crude aqueous extract was obtained by employing heat reflux with 10% (*w/v*) of the plant, and then liquid–liquid partitioning extraction was performed using ethyl acetate and butanol. The ethyl acetate fraction showed GI_50_ values ranging from 59 to 205 µg/mL in four human tumor cell lines (NCI-H460, MCF-7, HepG2, and HeLa) and a pig liver cell primary culture (PLP2, non-tumor cells) [42]. These values are in the range of those observed in the present study.

#### 3.2.2. Anti-Inflammatory Activity

The anti-inflammatory activity was assessed by evaluating the inhibition of NO production in LPS-stimulated RAW 264.7 mouse macrophage cells. The results, expressed as the concentration of extract required to inhibit 50% of NO production (IC_50_ values), are summarized in Table 3. Among the plants studied, *A. graveolens* demonstrated the strongest anti-inflammatory potential, with the lowest IC50 values ranging from 15 to 78 µg/mL. Conversely, the MAC and PEF extracts of *H. scoparium* and the PEF extract of *R. chalepensis* showed no significant ability to inhibit NO production in the applied cell-based assay (IC_50_ > 400 µg/mL). However, the MAC extract of *R. chalepensis* displayed notable anti-inflammatory activity with an IC_50_ of 77 μg/mL (Table 3).

The anti-inflammatory capacity of *R. chalepensis* was previously evaluated by nitrite quantification. For this, IFN-γ/LPS induced RAW 264.7 murine macrophages were employed, and the results revealed that *R. chalepensis* methanolic extract obtained by the soxhlet system showed 31% inhibition at 600 mg/L. In addition, these authors observed that the content of tannins and flavonoids was correlated with the anti-inflammatory activity [43]. Also, a previous [33] published manuscript revealed that the anti-inflammatory potential of *A. graveolens* aerial parts and callus aqueous extracts obtained by infusion process was tested through carrageenan-induced edema test. The results revealed that *A. graveolens* aerial parts extracts produced the highest anti-inflammatory effect (73%) at 100 mg/mL concentration. Thus, these results proved that *A. graveolens* extracts could be a potential source of anti-inflammatory agents. In another study, *H. scoparium* hydroalcoholic extracts obtained by sonication were evaluated for their anti-inflammatory activity by the 5-lipoxygenase assay. The authors reported that several compounds are related to the ability to inhibit lipoxygenase, namely flavonoids. However, the authors suggest that other compounds, such as phenolic acids or terpenoids, could also be able to inhibit the 5-lipoxygenase [41].

#### 3.2.3. Antioxidant Capacity

The TBARS method evaluates the capacity for preventing lipid peroxidation. All tested plant extracts showed antioxidant capacity (Table 3). *A. graveolens* MAC extract showed the greatest antioxidant capacity with an IC_50_ of 17 µg/mL. This IC50 was lower than that observed in the PEF extract (IC_50_ of 51 µg/mL). In the case of *H. scoparium*, both MAC and PEF extracts showed similar antioxidant properties, with IC50 values of 44.4 and 35.6 µg/mL, respectively. Finally, *R. chalapensis* was the plant that exhibited the lowest capacity for preventing lipid oxidation, with the IC_50_ lower in the PEF extract (50.5 µg/mL) compared to the MAC extract (79.5 µg/mL).

Contrary to these results, in a previous study, the TBARS showed an IC_50_ of 1925 µg/mL in *H. scoparium* collected from Morocco. In this case, the hydroalcoholic extracts were obtained by sonication. This value was higher in comparison to the present study [44]. The differences observed may be due to different techniques and the type of solvent used for bioactive compound extraction. Moreover, they suggested that flavonoid contents may prevent lipid peroxidation, although a correlation between phenol and flavonoid contents and TBARS values was not found [44]. A slightly lower antioxidant capacity was also reported in a previous study performed in extracts obtained from Algerian plants by infusion preparation, including some of the studied in the present study. In the case of *H. scoparium* and *A. graveolens*, the EC_50_ obtained was 164 and 139 µg/mL, respectively [28].

The antihemolytic activity assay (OxHLIA) to check the antioxidant capacity of the extracts revealed that *H. scoparium* was the plant with the highest antioxidant capacity with an IC_50_ of 6 µg/mL in the extract obtained by PEF and 19 µg/mL in the MAC extraction. In the case of *R. chalapensis*, the extract obtained employing MAC extraction showed the highest antioxidant capacity (IC_50_ 12 µg/mL) compared to that obtained by PEF (IC_50_ 67 µg/mL). Finally, the lowest antioxidant capacity was observed in the extract obtained from *A. graveolens* after MAC extraction (IC_50_ 88 µg/mL). In the case of *A. graveolens* PEF extract, the IC_50_ was 25 µg/mL (Table 3).

Since there is little information available about the antihemolytic activity assays of the spices studied in the present study, the results have been compared with other Algerian medicinal plants. In contrast to our results, [45] evaluated the antihemolytic activity of *Thymus algeriensis* extracts in petroleum ether, chloroform, and n-butanol solvents using the erythrocyte osmotic fragility model and found that the *n*-BuOH extract had a lower antioxidant capacity with an IC_50_ for hemolysis inhibition concentration of 322.85 ± 0.87 μg/mL. However, the antihemolytic activity was not measured kinetically by these authors, unlike the OxHLIA assay performed in this work. In another study, the OxHLIA assay performed on the butanolic fraction obtained after maceration extraction of the aerial parts of the Algerian plant (*Astragalus gombiformis Pomel*) showed an EC_50_ of 1643.78 µg/mL, with a lower antioxidant capacity than that observed in the present study. These authors also showed that the hemolytic activity of this extract was dose-dependent, increasing with increasing extract concentration (125, 250, 500, and 1000 μg/mL). In the present study, the extract concentrations tested were 125–8000 μg/mL [46]. So, the results obtained in the present study revealed a potential antioxidant capacity of the extracts obtained from the medicinal plants studied.

#### 3.2.4. Antibacterial Activity

The antibacterial activity of *A. graveolens*, *H. scoparium*, and *R. chalepensis* extracts against Gram-negative and Gram-positive strains are presented in Table 4. Regarding Gram-negative bacteria, *P. aeruginosa* and *S. enterocolitica* constitute the most resistant strains with minimum inhibitory concentration (MIC) values of 10 or >10 mg/mL (maximum concentration tested) for all medicinal plant extracts obtained by both PEF or MAC, with the exception of extracts obtained from *H. scoparium* under MAC with MIC values of 5 and 2.5 mg/mL for *P. aeruginosa* and *S. enterocolitica*, respectively. In the rest of the Gram-negative strains evaluated, MAC extracts of all tested plants seem to present a greater activity than PEF extracts against bacterial growth with MIC values ranging from 1.25 to 10 mg/mL, although in some cases, the same MIC value was obtained under MAC and PEF procedures (Table 4).

Concerning Gram-positive bacteria, *H. scoparium* extracts obtained by both PEF and MAC extraction resulted in the most effective bacterial growth inhibition, with MIC values ranging from 1.25 to 5 mg/mL, as well as *R. chalepensis* MAC extract with MIC (0.6–5 mg/mL), showing a great antibacterial capacity. In this case, better results were observed employing maceration in comparison with PEF extraction, where MIC values obtained were higher than the maximum concentration assayed (>10 mg/mL) (Table 4).

Among all strains tested, *Y. enterocolitica* and *S. aureus* constitute the most sensitive bacteria to Algerian plant extracts. Although interesting bacteriostatic activity was observed for the tested plant extracts, the same extracts did not present bactericidal activity at the maximum concentration tested.

Other authors have reported a higher antimicrobial capacity of *R. chalepensis*. The ethanolic extracts at different concentrations (1.56, 3.125, 6.25, 12.5, 25, 50, 100, and 200 μg/mL) showed robust antimicrobial activity against *Proteus penneri* and *S. aureus* with MICs of 12.5 and 25.0 μg/mL, respectively. This ethanolic extract of *R. chalepensis* showed the highest MIC (50 μg/mL) against *E. coli* [47]. In contrast, another study reported good antibacterial activity of the aqueous extract of *H. scoparium* leaves at higher concentrations tested (25 mg/mL) [48].

Similar antimicrobial properties to those observed in the present study were reported for other Algerian plants. For instance, leaf dichloromethane extracts of *Zizyphus lotus* L. showed interesting antimicrobial activities against *E. coli*, methicillin-sensitive *S. aureus*, and *Staphylococcus epidermis*, with MIC values ranging from 1024 to 2048 µg/mL [49]. In another study, *Thymus algeriensis* purified extracts showed an antibacterial effect against five bacterial strains. In this regard, the methanolic fraction obtained by reversed solid phase cartridge showed antibacterial potential against *E. coli*, *Proteus mirabilis*, *Salmonella typhimurium*, *Micrococcus luteus*, and *B. cereus* with MIC values ranging from 2 to 9 mg/mL [50]. These authors attributed the antibacterial properties to rosmarinic acid, kaempferol, and eriodictyol derivatives phenolic compounds.

Other authors reported higher antibacterial potential in other Algerian plants. The methanolic extracts of *Hedera helix*, *Pistacia lentiscus*, *Olea europaea*, and *Ziziphus lotus* obtained under refluxed employing a Soxhlet’s extractor, demonstrated antibacterial activity against various bacteria, including *E. coli*, *P. aeruginosa*, and *S. aureus*. The MIC values for Gram-positive bacteria ranged from 3 to 390 µg/mL, while those for Gram-negative bacteria were between 48 and 1600 µg/mL. These authors further proposed that the antibacterial effects of these methanolic plant extracts are linked to the phenolic compounds they contain, which exhibit distinct interactions with protein-associated polyamide polymers. In this sense, phenolic compounds could exert antimicrobial activity through iron deprivation or hydrogen bonding with vital proteins such as microbial enzymes. In addition, tannins are susceptible to polymerization through oxidation reactions in the air. Thus, oxidative condensation of phenols could lead to microbial damage [51].

#### 3.2.5. Antifungal Activity

Concerning antifungal activity, the extracts obtained from the plants studied in this work (*A. graveolens*, *H. scoparium*, *R. chalepensis*) by PEF extraction showed inhibitory activity against *Aspergillus brasiliensis* with MIC value at the maximum concentration assayed (10 mg/mL), unlike the extracts obtained by MAC that did not present fungistatic activity at the maximum concentration tested. Regarding Aspergillus fumigatus, MIC values were established at the maximum concentration assayed (10 mg/mL) for all the extracts tested, except for the MAC extract obtained from *R. chalepensis* with a MIC of 5 mg/mL. *R. chalepensis* MAC extract also showed promising results against the growth of several bacterial strains, as was reported before. In general, plant extracts tested in the present study were more effective in bacterial inhibition than in the fungal one (Table 4).

In a previous study, the essential oil derived from *A. graveolens* exhibited antifungal capability against *Aspergillus niger* and *Aspergillus flavus* with MIC around 24 µg/mL. However, the present study investigated the aqueous (PEF) and hydroethanolic (MAC) extracts of *A. graveolens* [52]. The antifungal activity might be ascribed to certain bioactive compounds such as α-thujone and carvacrol present in the essential oils. Another study reported that methanolic extracts of aerial parts of *H. scoparium* inhibited mycelial growth of *Aspergillus favus* by 65% [53]. The antifungal capacity of medicinal plants hydroethanolic extracts could be attributed to the presence of some compounds such as tannins, flavonoids, saponins, steroids, and alkaloids [54].

## 4. Conclusions

This research investigated three medicinal plants native to Algeria (*A. graveolens*, *R. chalepensis*, and *H. scoparium*) to assess their phenolic profiles and biological activities, including antioxidant, cytotoxic, antimicrobial, and anti-inflammatory properties. A total of 18 phenolic compounds were identified, including derivatives of caffeoylquinic acid, isorhamnetin, and quercetin. MAC proved more effective for extracting phenolic compounds than PEF. Specifically, the hydroethanolic MAC extract of *A. graveolens* displayed notable anti-inflammatory and cytotoxic activities, particularly against CaCo-2, MCF-7, and AGS tumor cell lines. Additionally, *A. graveolens* PEF aqueous extracts and *R. chalepensis* MAC hydroethanolic extracts demonstrated slightly higher anti-inflammatory activity.

In antioxidant assays, *H. scoparium* PEF extract exhibited the strongest activity, with an IC_50_ value of 6 µg/mL, followed by its MAC counterpart at 19 µg/mL. Both methodologies showed antioxidant potential across all plants studied. The antimicrobial evaluation revealed that MAC extracts were generally more effective against bacterial strains, although PEF extracts showed superior antifungal activity against *A. brasiliensis.* It is important to note that due to the nature of each technique, different extraction solvents were used, ethanol-water for maceration and water for PEF extraction, so differences observed in biological activities between the extraction techniques studied may also be due to the different solvents employed.

This work highlights the potential of PEF as a green and sustainable technology for extracting bioactive compounds from plant materials. While MAC often yielded better results, PEF demonstrated comparable performance in several assays, offering an environmentally friendly alternative by eliminating the need for organic solvents.

## Figures and Tables

**Table 1:** Retention time (Rt), wavelengths of maximum absorption in the visible region (λ_max_), mass spectral data, and identification of the phenolic compounds present in the MAC and PEF preparations of *A. graveolens*, *R. chalepensis*, and *H. scoparium*.

Peak (Identified Signals)	Rt (min)	λ_max_ (*nm*)	[M-H] (*m*/*z*)	MS^n^ (*m*/*z*)	Tentative Identification
*Asteriscus graveolens*					
1	6.17	320	707	353 (100), 191 (53)	5-*O*-Caffeoylquinic acid
2	8.72	327	593	503 (29.2), 474 (24), 473 (100), 383 (32.4), 353 (88.3)	Apigenin-di-*C*-glucoside
3	13.52	325	489	467 (100), 285 (32)	Luteolin-*O*-acetylhexoside
4	15.61	294	449	287 (20), 269 (100), 225 (2), 209 (2), 151 (27)	Eridictyol-*O*-hexoside
5	16.55	333	609	301 (100)	Quercetin-3-*O*-rutinoside
6	17.22	353	477	301 (100)	Quercetin-3-*O*-glucuronide
7	19.26	328	515	353 (100), 335 (5), 191 (25), 179 (13), 135 (2)	3,5-*O*-Dicaffeoylquinic acid
8	19.81	330	549	505 (100), 463 (18), 301 (52)	Quercetin-*O*-malonylhexoside
9	20.61	356	491	463 (100)	Tricin-*O*-glucoside
*Ruta chalepensis*					
10	5.53	311	337	191 (11), 163 (100)	4-*p*-Coumaroylquinic acid
11	6.23	324	367	193 (100)	4-*O*-Feruloylquinic acid
12	8.62	325	593	473 (100), 353 (52)	Apigenin-6-*C*-glucose-8-*C*-glucose
13	10.0	330	593	473 (100), 353 (12)	Apigenin-8-*C*-glucose-6-*C*-glucose
14	11.9	327	507	269 (100)	Apigenin-*O*-glucuronyl-hexoside
15	12.4	328	959	735 (100), 529 (11), 511 (17)	Tri-sinapoyl-gentiobiose
16	14.1	332	611	301 (100)	Quercetin-*O*-deoxyhexosyl-hexoside
17	15.6	332	639	315 (100)	Isorhamnetin-*O*-di-hexoside
18	16.6	345	609	301 (100)	Quercetin-3-*O*-rutinoside
19	17.3	353	477	301 (100)	Quercetin-3-*O*-glucuronide
20	19.7	336	653	287 (100)	Eriodictyol-*O*-acetyl-di-hexoside
21	20.8	348	623	315 (100)	Isorhamnetin-3-*O*-rutinoside
22	21.9	330	753	529 (100), 223 (14)	1,2-Disinapoylgentiobioside
23	31.6	327	959	735 (100), 529 (12)	1,2,2′ -Trisinapoylgentiobiose
24	32.2	323	929	735 (16), 705 (100)	1,2-Disinapoyl-2-feruloylgentiobiose
25	39.1	326	723	529 (19), 499 (100), 233 (6)	Sinapoyl-feruloylgentiobiose
26	43.5	330	325	183 (100), 119 (11)	unknown
*Haloxylon scoparium*					
27	3.47	281	153	109 (100)	Protocatechuic acid
28	3.77	320	179	135 (100)	Caffeic acid
29	4.01	281	359	197 (100)	Glycosyringic acid
30	4.78	282	325	163 (100)	*p*-Coumaric acid hexoside
31	7.42	321	387	369 (25), 207 (100), 163 (49)	Caffeic acid acetylhexoside
32	9.83	342	785	623 (100), 315 (33)	Isorhamnetin-*O*-hexoside-*O*-deoxyhexosyl-hexoside
33	10.1	322	565	519 (68), 403 (100), 385 (22), 223 (41)	Caffeic acid-*O*-(sinapoyl-*O*-hexoside)
34	11.8	334	917	873 (55), 669 (100), 505 (10), 315 (21)	Isorhamnetin-*O*-hydroxyferuloylhexoside-*O*-malonylhexoside isomer I
35	12.7	333	917	873 (60), 669 (100), 505 (15), 315 (32)	Isorhamnetin-*O*-hydroxyferuloylhexoside-*O*-malonylhexoside isomer II
36	14.0	345	639	477 (28), 315 (100)	Isorhamnetin-*O*-dihexoside
37	14.6	340	741	609 (100), 301 (56)	Quercetin-*O*-pentoside-*O*-deoxyhexoside-hexoside
38	16.3	347	769	315 (100)	Isorhamnetin-*O*-di-deoxyhexosyl-hexoside
39	17.5	353	755	605 (46), 315 (100)	Isorhamentin-*O*-deoxyhexosyl-pentosyl-hexoside isomer I
40	18.4	350	755	315 (100)	Isorhamentin-*O*-deoxyhexosyl-pentosyl-hexoside isomer II
41	20.2	353	623	315 (100)	Isorhamnetin-*O*-deoxyhexoside-*O*-hexoside isomer I
42	20.8	350	623	315 (100)	Isorhamnetin-*O*-deoxyhexoside-*O*-hexoside isomer II
43	21.4	351	623	315 (100)	Isorhamnetin-3-*O*-rutinoside
44	22.0	345	477	315 (100)	Isorhamnetin-*O*-glucoside

MAC: maceration; PEF: pulsed electric field.

**Table 2:** Quantification (mg/g of extract) of the phenolic compounds tentatively identified in *A. graveolens*, *R. chalepensis*, and *H. scoparium* MAC and PEF extracts.

Compounds	Quantification (mg/g Extract)		*p*-Value
	MAC	PEF	
*Asteriscus graveolens*			
5-*O*-Caffeoylquinic acid ^1^	19.3 ± 0.3	5.83 ± 0.1	0.002
Apigenin-di-*C*-glucoside ^2^	1.1 ± 0.1	1.6 ± 0.1	0.008
Luteolin-*O*-acetylhexoside ^11^	0.74 ± 0.01	0.66 ± 0.01	0.01
Eridictyol-*O*-hexoside ^3^	0.023 ± 0.001	nd	0.007
Quercetin-3-*O*-rutinoside ^4^	0.163 ± 0.003	0.0026 ± 0.0001	0.004
Quercetin-3-*O*-glucuronide ^4^	4.9 ± 0.2	2.15 ± 0.01	0.01
3,5-*O*-Dicaffeoylquinic acid ^1^	3.6 ± 0.1	0.75 ± 0.01	0.006
Quercetin-*O*-malonylhexoside ^4^	0.81 ± 0.05	2.59 ± 0.03	0.0004
Tricin-*O*-glucoside ^4^	0.24 ± 0.02	0.018 ± 0.001	0.01
Total phenolic acids #	22.8 ± 0.4	6.6 ± 0.1	0.003
Total Flavonoids #	7.9 ± 0.1	7.0 ± 0.1	0.01
Total phenolic compound #	30.7 ± 0.4	13.6 ± 0.2	0.0004
*Ruta chalepensis*			
4-*p*-Coumaroylquinic acid ^1^	4.7 ± 0.1	4.7 ± 0.2	0.3
4-*O*-Feruloylquinic acid ^5^	1.7 ± 0.1	2.0 ± 0.1	0.08
Apigenin-6-*C*-glucose-8-*C*-glucose ^2^	5.3 ± 0.1	6.0 ± 0.1	0.006
Apigenin-8-*C*-glucose-6-*C*-glucose ^2^	1.7 ± 0.1	1.5 ± 0.1	0.05
Apigenin-*O*-glucuronyl-hexoside ^2^	1.224 ± 0.001	2.40 ± 0.03	0.005
Tri-sinapoyl-gentiobiose ^6^	0.48 ± 0.03	0.31 ± 0.01	0.02
Quercetin-*O*-deoxyhexosyl-hexoside ^4^	1.24 ± 0.02	0.92 ± 0.02	0.001
Isorhamnetin-*O*-di-hexoside ^4^	23.1 ± 0.1	1.38 ± 0.01	0.001
Quercetin-3-*O*-rutinoside^4^	9.9 ± 0.5	28.9 ± 0.5	0.0004
Quercetin-3-*O*-glucuronide^4^	2.16 ± 0.02	2.21 ± 0.01	0.06
Eriodictyol-O-acetyl-di-hexoside ^3^	5.4 ± 0.3	1.00 ± 0.03	0.01
Isorhamnetin-3-*O*-rutinoside ^4^	3.9 ± 0.1	2.16 ± 0.03	0.01
1,2-Disinapoylgentiobioside ^6^	0.54 ± 0.02	2.11 ± 0.02	0.0002
1,2,2′ -Trisinapoylgentiobiose ^6^	0.62 ± 0.03	0.90 ± 0.01	0.01
1,2-Disinapoyl-2-feruloylgentiobiose ^6^	4.6 ± 0.2	1.15 ± 0.01	0.01
Sinapoyl-feruloylgentiobiose ^6^	4.8 ± 0.1	0.22 ± 0.01	0.001
Unknown*	nq	nq	-
Total phenolic acids #	18 ± 1	11.4 ± 0.3	0.006
Total flavonoids #	54 ± 1	46.5 ± 0.3	0.03
Total phenolic compound #	72 ± 2	58.01 ± 0.04	0.03
*Haloxylon scoparium*			
Protocatechuic acid ^7^	12.8 ± 0.2	nd	0.002
Caffeic acid ^8^	6.2 ± 0.3	nd	0.01
Glycosyringic acid ^9^	73.9 ± 1.5	nd	0.004
*p*-Coumaric acid hexoside ^10^	25.6 ± 0.2	10.8 ± 0.3	0.0005
Caffeic acid acetylhexoside ^8^	6.0 ± 0.2	10.5 ± 0.1	0.005
Isorhamnetin-*O*-hexoside-*O*-deoxyhexosyl-hexoside ^4^	6.9 ± 0.4	nd	0.01
Caffeic acid-*O*-(sinapoyl-*O*-hexoside) ^8^	0.063 ± 0.002	nd	0.007
Isorhamnetin-*O*-hydroxyferuloylhexoside-*O*-malonylhexoside isomer I ^4^	0.79 ± 0.02	0.91 ± 0.02	0.01
Isorhamnetin-*O*-hydroxyferuloylhexoside-*O*-malonylhexoside isomer II ^4^	0.686 ± 0.001	0.733 ± 0.001	0.0004
Isorhamnetin-*O*-dihexoside ^4^	0.67 ± 0.01	0.716 ± 0.002	0.01
Quercetin-*O*-pentoside-*O*-deoxyhexoside-hexoside ^4^	1.01 ± 0.01	0.946 ± 0.03	0.1
Isorhamnetin-*O*-di-deoxyhexosyl-hexoside ^4^	1.48 ± 0.02	1.30 ± 0.02	0.008
Isorhamentin-*O*-deoxyhexosyl-pentosyl-hexoside isomer I ^4^	3.15 ± 0.03	2.84 ± 0.02	0.01
Isorhamentin-*O*-deoxyhexosyl-pentosyl-hexoside isomer II ^4^	1.27 ± 0.02	1.27 ± 0.01	0.4
Isorhamnetin-*O*-deoxyhexoside-*O*-hexoside isomer I ^4^	1.72 ± 0.02	1.75 ± 0.02	0.1
Isorhamnetin-*O*-deoxyhexoside-*O*-hexoside isomer II ^4^	0.89 ± 0.02	0.87 ± 0.02	0.1
Isorhamnetin-3-*O*-rutinoside ^4^	0.25 ± 0.01	0.46 ± 0.03	0.02
Isorhamnetin-*O*-glucoside ^4^	0.656 ± 0.001	0.736 ± 0.002	0.0004
Total phenolic acids #	125 ± 2	21.2 ± 0.4	0.003
Total flavonoids #	19.4 ± 0.3	12.5 ± 0.1	0.005
Total phenolic compound #	144 ± 2	33.8 ± 0.5	0.001

Nd: not detected; nq: not quantified; MAC: maceration; PEF: pulsed electric field; calibration curves used for each compound: 1. chlorogenic acid (y = 168823x − 161172; R^2^ = 0.9999, LOD = 0.20 µg/mL and LOQ = 0.68 µg/mL); 2. apigenin-6-*C*-glucoside (y = 107025x + 61531, R^2^ = 0.998; LOD = 0.19 µg/mL; LOQ = 0.63 µg/mL); 3. naringenin (y = 18433x + 78903, R^2^ = 0.9998; LOD = 0.20 μg/mL; LOQ = 0.64 μg/mL); 4. quercetin-3-*O*-glucoside (y = 34843x − 160173, R^2^ = 0.9999, LOD = 0.21 µg/mL; LOQ = 0.71 µg/mL) 5. ferulic acid (y = 633126x − 185462, R^2^ = 0.999, LOD = 0.20 µg/mL and LOQ = 1.01 µg/mL); 6. sinapic acid (y = 197.337x + 30036; R^2^ = 0.999; LOD = 0.17 μg/mL; LOQ = 1.22 μg/mL); 7. protocatechuic acid (y = 214168x + 27102, R^2^= 0.9999; LOD = 0.14 μg/mL; LOQ = 0.52 μg/mL); 8. caffeic acid (y = 388,345x + 406,369; R^2^ = 0.994; LOD = 0.78 μg/mL; LOQ = 1.97 μg/mL); 9. syringic acid (y = 53993x + 4671.4, R^2^ = 0.998; LOD = 0.50 µg/mL; LOQ = 0.98 µg/mL); 10. *p*-coumaric acid (y = 301950x + 6966.7, R^2^ = 0.9999, LOD = 0.68 µg/mL; LOQ = 1.61 µg/mL); and 11. luteolin-6-*C*-glucoside (y = 4087.1x + 72.589, R^2^ = 0.999, LOD = 0.86 µg/mL; LOQ = 1.67 µg/mL. * “Unknown” refers to compounds detected that could not be reliably identified based on our standards or the available literature. # The values represent the sum of concentrations (mg/g) for each compound family, determined using calibration curves and calculated from peak areas or signal intensities. Significant differences (*p* < 0.05) between extracts were assessed by Student’s *t*-test.

**Table 3:** Cytotoxic, antioxidant, and anti-inflammatory activities of the hydroethanolic extracts of *A. graveolens*, *H. scoparium*, and *R. chalepensis* employing pulsed electric field (PEF) and maceration (MAC) extraction procedures.

	*A. graveolens*		*H. scoparium*		*R. chalepensis*	
	MAC	PEF	MAC	PEF	MAC	PEF
*Cytotoxic activity (GI_50_, µg/mL)*						
AGS	17 ± 2 a	62 ± 2 b	156 ± 14 c	111 ± 8 d	119 ± 2 d	>400 e
CaCo-2	56 ± 3 a	85 ± 4 b	275 ± 12 c	266 ± 10 d	45 ± 3 e	>400 f
MCF-7	43 ± 4 a	69 ± 1 b	250 ± 5 c	242 ± 22 c	39 ± 4 a	>400 d
VERO	43 ± 4 a	74 ± 7 b	146 ± 6 c	81 ± 8 b	136 ± 4 d	>400 e
PLP2	13 ± 1 a	56 ± 2 b	209 ± 8 c	201 ± 18 c	55 ± 5 b	>400 d
*Anti-inflammatory activity (IC_50_, µg/mL)*						
RAW 264.7	15 ± 1 a	78 ± 2 b	>400 c	>400 c	77 ± 1 b	>400 c
*Antioxidant activity (IC_50_, µ* *g/mL)*						
TBARS	17 ± 4 e	51 ± 1 b	44.4 ± 8 ab	35.6 ± 1 a	79.5 ± 14 d	50.5 ± 1 b
OxHLIA	88 ± 3 f	25 ± 2 d	19 ± 1 c	6 ± 1 a	12 ± 1 b	67 ± 3 e

Results are presented as the mean ± SD. MAC: maceration; PEF: pulsed electric field; GI_50:_ concentration that inhibited 50% of the cell’s proliferation. GI_50_ values for Ellipticine: 1.23 ± 0.03 µg/mL (AGS), 1.21 ± 0.02 µg/mL (Caco-2), 1.02 ± 0.02 µg/mL (MCF-7), 1.41 ± 0.06 µg/mL (Vero), 1.4 ± 0.1 µg/mL (PLP2). IC_50_ values for Dexamethasone: 6.3 ± 0.4 µg/mL (RAW 264.7). Positive control for antioxidant activity: Trolox: 21.8 ± 0.2 µg/mL. Different letters in each row correspond to significant differences (*p* < 0.05) among several extracts for each cell line.

**Table 4:** Antibacterial and antifungal activities of extracts obtained from Algerian medicinal plants employing PEF and MAC extraction techniques.

	*Haloxylon scoparium*				*Asteriscus graveolens*				*Ruta chalepensis*			
	PEF		MAC		PEF		MAC		PEF		MAC	
Antibacterial activity (mg/mL)												
	MIC	MBC	MIC	MBC	MIC	MBC	MIC	MBC	MIC	MBC	MIC	MBC
Gram-negative bacteria												
*Enterobacter Cloacae*	5	>10	5	>10	10	>10	5	>10	10	>10	5	>10
*Escherichia coli*	5	>10	2.5	>10	10	>10	10	>10	10	>10	10	>10
*Pseudomonas aeruginosa*	>10	>10	5	>10	>10	>10	10	>10	>10	>10	10	>10
*Salmonella enterica*	10	>10	2.5	>10	>10	>10	10	>10	10	>10	10	>10
*Yersinia enterocolitica*	2.5	>10	2.5	>10	10	>10	2.5	>10	2.5	>10	1.25	>10
Gram-positive bacteria												
*Bacillus cereus*	5	>10	5	>10	10	>10	2.5	>10	>10	>10	0.6	>10
*Listeria monocytogenes*	5	>10	2.5	>10	10	>10	5	>10	>10	>10	5	>10
*Staphylococcus aureus*	2.5	>10	1.25	>10	>10	>10	5	>10	>10	>10	2.5	>10
Antifungal Activity mg/mL												
	MIC	MFC	MIC	MFC	MIC	MFC	MIC	MFC	MIC	MFC	MIC	MFC
*Aspergillus brasiliensis*	10	>10	>10	>10	10	>10	>10	>10	10	>10	>10	>10
*Aspergillus fumigatus*	10	>10	10	>10	10	>10	10	>10	10	>10	5	>10

MAC: maceration; PEF: pulsed electric field; MIC: minimum inhibitory concentration; MBC: minimum bactericidal concentration; MIC and MBC values for positive controls: streptomycin (0.007–0.06 mg/mL), ampicillin (0.15-0.63 mg/mL), and methicillin (0.007 mg/mL). MFC: minimum fungicidal concentration; MIC and MFC values for positive controls: Ketoconazole (0.06–0.125 mg/mL)/(0.5–1 mg/mL).

## Data Availability

The original contributions presented in the study are included in the article, further inquiries can be directed to the corresponding author.
